# Digital Twin of Coal Mine Rescue Robot—Research on Intelligence and Visualization

**DOI:** 10.3390/s26092840

**Published:** 2026-05-01

**Authors:** Shaoze You, Menggang Li, Baolei Wu, Jun Wang, Chaoquan Tang

**Affiliations:** 1 School of Information and Control Engineering, China University of Mining and Technology, Xuzhou 221116, China; youshaoze@cumt.edu.cn; 2 Jiangsu Collaborative Innovation Center of Intelligent Mining Equipment, China University of Mining and Technology, Xuzhou 221008, China; sallylmg@cumt.edu.cn (M.L.); tangchaoquan@cumt.edu.cn (C.T.); 3 School of Mechatronic Engineering, China University of Mining and Technology, Xuzhou 221116, China

**Keywords:** coal mine, rescue robots, path planning, autonomous navigation, digital twins

## Abstract

Mine disasters require urgent lifeline setup in confined tunnels, but manual rescue in unstable accident zones carries huge safety risks. Coal mine rescue robots (CMRRs) have become key equipment to replace manual rescue. However, traditional remote-controlled CMRRs suffer from low autonomy and weak environmental perception capability, which have become critical bottlenecks for field application. As an emerging technology in the mining field, digital twin enables high-precision virtual-real mapping and on-site operation guidance, providing a novel solution to the above problems. To realize autonomous navigation and digital twin visualization of the CMRR, this paper first carries out targeted hardware retrofits on the CMRR platform, upgrades environmental perception, communication transmission and motion control modules, and lays a solid hardware foundation for subsequent algorithm design and system implementation. Aiming at the complex post-disaster underground environment, a digital twin-integrated CMRR system is constructed. For intelligent autonomous navigation, this study investigates a 3D point cloud–based autonomous navigation framework and proposes a slope-fitting method as well as a maximum arrival probability obstacle avoidance method based on Bézier curve trajectories. For environmental visualization, a digital twin interactive interface is built to monitor gas and other environmental parameters in real time, and accurately reconstruct underground roadway structures based on point cloud data. This design not only ensures the robot’s autonomous obstacle avoidance but also helps rescuers grasp underground conditions in advance. Field tests in a simulated post-disaster mine with complex terrain show that the system can stably complete autonomous navigation tasks, maintain stable motion control under dynamic interference, and provide accurate and reliable environmental data for rescue decisions, verifying its feasibility and effectiveness in harsh mine rescue scenarios.

## 1. Introduction

Coal remains one of the world’s most abundant energy sources, with its production efficiency and scale playing a critical role in supporting a nation’s industrial infrastructure. China is the world’s largest coal producer (approximately 47.5%) and holds the third-largest coal reserves globally. However, among major coal-producing countries, China faces the most challenging mining conditions and the highest frequency of mining disasters [1]. Statistics indicate that gas and dust explosions are the most prevalent coal mine accidents, responsible for over 60% of fatalities in coal mining incidents. Following such explosions, the post-accident site becomes an unstructured environment characterized by unknown conditions: it is filled with toxic gases, and obstacles are collapsed or displaced. These factors not only restrict the rapid movement of human rescuers but also pose severe threats to their safety. Thus, deploying robots to replace human workers in hazardous post-disaster environments has emerged as a key trend in mine disaster response [2].

The primary task of a coal mine rescue robot (CMRR) is to rapidly and timely enter disaster-stricken areas. It needs to detect key environmental and situational parameters, which include concentrations of various gases, temperature, humidity, locations of trapped personnel, and the structural morphology of post-disaster scenes, and then transmit this information to the rescue command center via data, voice, and image modalities [3]. Currently, most underground search, rescue, and detection robots rely on teleoperation for exploration tasks; however, the complex post-disaster environment poses significant challenges to human operators. By contrast, ground-based unmanned technologies have achieved maturity and commercialization, and specialized equipment competitions such as the DARPA SUBT Underground City Challenge have emerged as drivers for technological advancement. Concurrently, core technologies, including autonomous robot navigation and swarm planning, continue to develop [4]. Thus, transferring and adapting these advanced surface-based navigation technologies to underground coal mine environments is critical for the development of intelligent CMRRs. Notably, post-disaster underground environments undergo dramatic changes, which require robots to capture large volumes of unknown information. Therefore, an intuitive visual operation system is necessary to integrate and present all key information to operators, and this requirement is essential for guiding subsequent rescue operations.

The digital twin technology makes full use of physical models, sensor data, and other information to perform the mapping of physical and state quantities in the virtual space representing the real world. It can reflect the full working cycle of the corresponding physical equipment processes [5]. It has great artificial intelligence advantages in solving tasks in the advanced manufacturing, energy industry, and other fields [6]. The definition and application of digital twin technology are consistent with localization mapping, autonomous navigation, and its visualization in the field of robotics. This paper aims to apply the digital twin technology to the rescue of coal mines and explore it based on CMRR.

**Figure 1:**
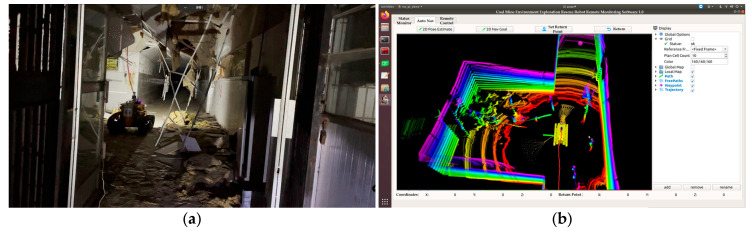
Real scenes with digital twin display interface. (**a**) Collapsed chamber. (**b**) Digital twin system autonomous navigation interface.

This study is an extension of a previous work, which was on a CMRR that obtained the Chinese coal mine safety certification. It upgrades and modifies the originally developed CMRR ontology [2]. The novel developed robot is named CUMT-V(C). The digital twin of the CMRR was built to display environmental information on the monitoring and management interface and to use this information to guide the navigation and obstacle avoidance of the robot in the real world (shown in Figure 1). The contributions of this study are summarized as follows:

1. A new digital twin interface is designed with a fast navigation system in the unstructured underground environment. It enables navigation and obstacle avoidance directly on the laser point cloud map, as well as the accurate mapping and presentation of the point cloud map to the real environment.
2. Two technical improvements for trajectory planning are proposed: first, Bézier curves are used instead of spline curves to generate offline local trajectory lookup tables; second, Voronoi constraint terms are incorporated into the optimal trajectory selection function. These optimizations enhance adaptability to navigation scenarios such as mine shafts and post-disaster environments.
3. A terrain analysis method based on a slope fitting strategy is proposed. It enables the calculation of terrain slopes using point cloud data acquired by LiDAR and adjusts passability parameters in accordance with the climbing capacity of the CMRR to ensure the feasibility of the CMRR’s slope navigation in underground coal mine scenarios.
4. Field experiments verify the effectiveness of this digital twin and robot navigation system in practical applications, enabling robots to avoid obstacles, navigate tight and cluttered spaces, and display real-time status. This work frees CMRR from wired transmission constraints, transforms them from full manual remote control to autonomous mobile with target points, and significantly reduces manual intervention, thereby enhancing their flexibility and search and rescue efficiency.

## 2. Related Works

### 2.1. Digital Twin in Coal Mines

In contrast to the meta-universe, which is simply presented in the virtual world [7], the digital twin is not just a digitalization and visualization, but it represents a real mapping of the real world to the virtual world. On the one hand, satellite remote sensing and laser scanning are used to obtain the physical data of the real world. On the other hand, 3D reconstruction, semantic modeling and simulation are used to replicate into various virtual worlds. A real-time interconnection is established between the physical world and the digital world. The concept of digital twin technology was first introduced by Grieves and was used in NASA’s Apollo research project [8]. The digital twin of coal mining is the digitization of information from coal mine geological models, hydrological models, and roadway models to enable the digitization and real-time interaction in the mining process using digital models, simulation, analysis, control, and associated feedback [9]. Xie et al. [10] used virtual reality (VR) technology to develop a digital twin application for the virtualization and digitization of a hydraulic support at a coal mining face. Zhang et al. [11] proposed an integration scheme of an intelligent mine based on a digital twin and built a digital twin model of the mine in Unity. It uses digital twin + 5G technology to achieve full domain awareness and data-driven and -assisted decision-making. Li et al. [12] proposed the use of an augmented reality software with a physics engine to simulate the behavior of real header mining equipment in contact with the coal seam and to guide the real-world implementation of the working posture and construction methods of coal mine header mining equipment.

### 2.2. Status of the Development of Coal Mine Robots

Many research institutions conducted studies on intelligent robots for underground mining. Neumann et al. [13] developed Barney, a mobile robot for underground environmental mapping in coal mines, which was driven by the Robot Operating System (ROS), and a Velodyne LIDAR was deployed on the robot for environmental sensing. However, Barney is not explosion-proof, and it was only used in a highway tunnel 2.4 km in length. Lösch et al. [14] developed Julius, a robot for underground mine detection, which was equipped with a binocular camera, IMU, and LIDAR, and teleoperated by ROS. In contrast to Barney, Julius is equipped with a UR5 robot arm to perform grasping tasks. Grehl et al. [15] developed Alexander, a typical mobile robot for underground mine mapping and monitoring, and successfully mapped coal mine tunnels in 2D and 3D in two mining areas. Miller et al. [16] developed a quadruped robot hardware and software system with modules for localization mapping, path planning, and target recognition, which was successfully applied to DAPAR Subterranean Challenge (SubT). However, none of the above robots were designed with explosion-proofing in mind.

Many studies on CMRRs were conducted. However, most of them are teleoperated. The Ralter is the world’s first CMRR. It was created in the USA and field tested in the 1990s [17]. Due to the performance limitations of the equipment, it did not complete the rescue mission. However, it has a reference value for the development of the subsequent CMRRs. The Groundhog fully autonomous mine detection robot [18], the *V2* coal mine environmental detection robot [19], and the Gemini-Scout environmental detection robot [20] were then launched by research institutions in the USA. Australian research institutions developed the massive Numbat remote-controlled detection robot [21] and the TeleRescuer coal mine detection robot, which carries a 3D LIDAR [22].

**Figure 2:**
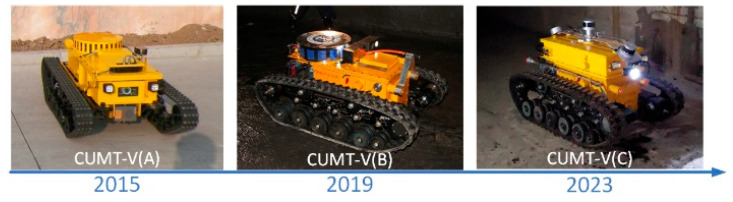
CUMT-V series coal mine rescue robots. CUMT-V(A) completed the design of the robot’s locomotion mechanism and explosion-proof structure. CUMT-V(B) completed the implementation of the robot’s environmental detection function, design of the communication system and rescue mechanism, structural optimization, and explosion-proof certification. CUMT-V(C) completes the study on robot perception, localization, intelligence, and digital twin visualization.

China University of Mining and Technology developed China’s first CMRR (CUMT-I) in 2006 [23]. Several types of CUMT series were then developed, with improvements made in the design of the CMRR’s locomotion mechanism [24], explosion-proof optimization [25], mapping and localization [26,27]. The CUMT-V(B) CMRR was demonstrated on site in Tashan coal mine in Datong, Shanxi, in 2016. It achieved good application results, providing valuable experience for the development and application of CMRRs [2]. Figure 2 illustrates the development of the CUMT-V series robots. Harbin Institute of Technology has developed several mobile robots for mine rescue applications, with the representative ones being the child–mother rescue robot [28] and the MINBOT series of robots [29], and has completed autonomous navigation experiments in underground buildings in subsequent work [30]. Beijing Institute of Technology has developed the tracked-legged mining search and rescue robot [31] and the MSRBOTS mine breaking robot [32].

### 2.3. Path and Trajectory Planning

Path planning aims at achieving a discrete line of collision-free position points on differently normalized maps, while trajectory planning ensures the continuity and kinematic feasibility of robot’s control space. The Hybrid A* algorithm, which can plan operations on unknown unstructured terrain, was proposed by Stanford University and successfully used on the Ackerman steering model vehicle in the DARPA Challenge [33]. ETH Zurich proposed a 3D unstructured terrain navigation framework that uses initial trajectory generation based on sampling and optimization by evaluating the point cloud terrain, which allows the planning of ground vehicles directly on a disordered point cloud [34]. NTNU proposed a graph-based autonomous exploration framework and further studied the rapid area exploration method of deep learning, which performed the autonomous exploration of underground caves by legged robots combined with UAVs [35]. CMU proposed a planning algorithm that maximizes the arrival probability model and uses an offline lookup table form to perform planning between ground vehicles and UAVs on dense point cloud maps [36]. HKUST proposed an online navigation framework for UAVs that establishes flight corridors through distance constraints, employs Bézier curves for trajectory planning directly on point cloud, and allows the collaborative multi-aircraft exploration in limited areas [37,38]. CUMT proposed a trajectory planning method based on the MINCO trajectory and safety corridor constraints constructed with underground environmental constraints [39]. ZJU Fast Lab proposed a series of motion planning frameworks, such as the optimization of demonstration reproduction trajectories [40] and swarm planning [41]. MIT proposed a fast-planning framework for unknown environments based on topological complementary structure representation of feasible regions. It can be used for UAVs and ground robots to perform obstacle avoidance at high speeds and allows the collaborative multi-aircraft planning for up to 32 UAVs [42,43].

Many current coal mining vehicles can be navigated in normalized maps [44,45]. Although normalized maps are efficient in data storage and searching, they do not reflect well the post-disaster roadway morphological features in complex scenarios after underground disasters. On the contrary, the direct navigation of the point cloud can solve this problem. Point cloud maps do not require configuration space discretization or complex maintenance, and studies have also been conducted on point cloud map-based navigation [34,36,37]. Therefore, the use of point cloud map information for autonomous navigation of coal mine search and rescue robots reduces the loss of efficiency due to map maintenance. Moreover, the dense point cloud map can directly reproduce the shape of the post-disaster scene and the degree of destruction.

## 3. System Overview

### 3.1. Hardware Architecture

**Figure 3:**
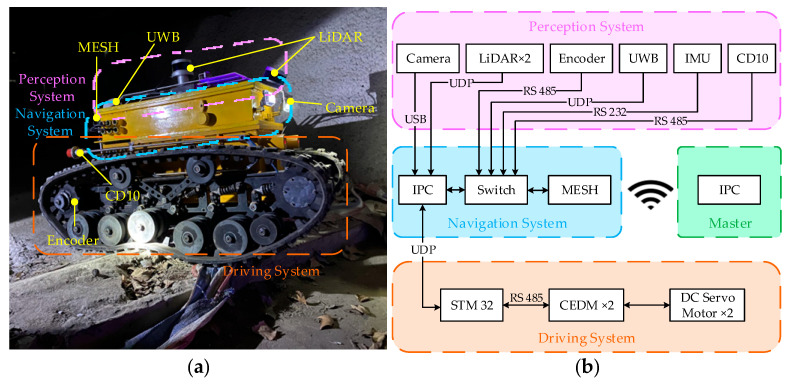
The CMUT-V(C) platform and the hardware system architecture diagram. (**a**) shows the location of some key sensor hardware. IMU, IPC, switches, and other equipment are loaded in the explosion-proof case of the navigation system. (**b**) illustrates the communication architecture of the hardware system. IPC: Industrial personal computer; CEDM: Control element drive motor; UWB: Ultra-wide band; IMU: Inertial measurement unit; LiDAR: Light detection and ranging; UDP: User datagram protocol.

The CUMT-V series robots mainly tackled the locomotion mechanism, explosion-proof structure (CUMT-V(A)), and rescue detection function (CUMT-V(B)) in the early stage [2]. This study draws on the research experience in explosion-proof design with coal mine safety certification of the CUMT-V(B) platform, redesigns the drive system, communication mode and environmental perception module, and conducts intelligent and visual research on the robot based on the newly developed CUMT-V(C) platform. The distribution of the robot hardware areas and the combination of hardware systems are shown in Figure 3. The performed modifications are summarized as follows:

1. The fiber-optic disk, Wi-Fi antenna, IR explosion-proof camera on the top of the robot, and the relay node on the back of the robot are canceled. The firetide© HotPort 7012 MESH node, which meets the coal safety requirements, is used instead of the original Wi-Fi relay module based on single node transmission, which improves the communication performance and increases the speed.
2. The electrical control box is redesigned and added to the upper part of the robot’s original box to ensure accurate mapping and autonomous navigation algorithms. The box is designed to be explosion-proof according to GB3836.2-2021 [46], which ensures the high overall explosion-proof performance of the robot.
3. A Nuvo-7160 GC series industrial control computer (© Neousys Technology, Shenzhen, China) with a rugged case and high vibration resistance is the main computing module of the robot. The STM32F407 microcontroller development board (Guangzhou Xingyi Electronic Technology Co., Ltd., Guangzhou, China) with ARM 32-bit Cortex™-M4 core is used to control the motors and receive encoder information for the robot’s lower computer.
4. A bracket is designed to mount and secure the sensor. Two Velodyne VLP-16 Puck LITE 3D LiDARs (© Velodyne, San Jose, California, USA) are mounted above the bracket. The first one is horizontally mounted to detect the surroundings, while the second one is mounted at an angle of 40° to detect the roof profile. Note that both can be replaced with intrinsically safe LiDARs at a later stage.
5. A ZED mini (© Stereolabs, New York, USA) and a RealSense D435i binocular camera (© Intel Corporation, Santa Clara, California, USA) are mounted in the front and upper part of the bracket to replace the original infrared camera, which is used to perform target identification of casualties and hazardous materials. The front of the robot is equipped with an array of detachable coal mine intrinsically safe LED lights with flood and high beam combination to ensure a high-quality video imaging.
6. A PulsOn 440 UWB (© Aunion Tech Co., Ltd., Shanghai, China) auxiliary positioning antenna is added to the rear of the mount, while the positioning chip is placed in the electrical box in the upper part of the robot and led through an intrinsically safe signal line. This allows the in-region positioning through the established UWB anchor node.
7. The original 4WD system (4 × 500 W) with slider shift mechanism is removed and replaced with a rear-drive dual DC motor system (2 × 1000 W). A hollow shaft encoder is added to provide real-time feedback of the crawler speed.

All hardware modifications strictly follow GB3836.2-2021 explosion-proof standards and fully inherit the safety certification of the CUMT-V(B) platform. The robot has passed 24-h continuous operation tests with stable performance of sensors, computing unit, and driving system. In addition, the robot is equipped with independent power management systems in upper and lower cabins, which can monitor the working condition and health state of battery cells in real time to guarantee stable power operation. Table 1 compares the mechanical model parameters and performance parameters of the CUMT-V(C) platform with the legacy CUMT-V(B) platform.

### 3.2. Software Architecture

**Figure 4:**
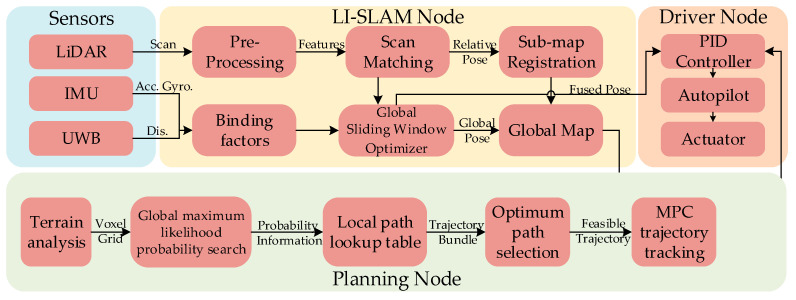
The software architecture of the CUMT-V(C) platform. The environmental information acquired in the SLAM node consists of three sensors. In this paper, UWB is not used as an optional device. The path planning node contains three sub-nodes: global planning, local planning, and trajectory tracking. The local planning is directly queried by the offline lookup table and the trajectory tracking is controlled by the MPC algorithm to fit the robot speed.

Figure 4 shows the complete software architecture of the CUMT-V(C), from sensing to planning and control. To perform these functions, the robot should first register the data from 3D LiDAR and binocular cameras using the SLAM algorithm in order to obtain a global map of the registered point cloud [26]. The robot’s positional prediction is then performed by fusing the IMU with the point cloud registration data [47]. Afterward, on the disordered point cloud, a local planning method using the Bézier curve offline trajectory lookup table and an improved optimal path selection method based on Falco [36] are proposed to develop the CMRR path planning algorithm. Finally, a feasible reference path is obtained. The generated trajectory is sent to the MPC [48] path tracking node to accurately track the path trajectory. The robot chassis motion control is then achieved through the PID output control of the motor driver. The entire navigation planning process, from the instant sensing to the extraction of the collision-free trajectories and the generation of the best trajectory, is performed in the robot-equipped IPC in a few tens of milliseconds.

At the lower-level controller of CUMT-V(C), the motion control node is programmed in the C language. It is used to transmit and receive input/output (I/O) information with the STM32. Additionally, an encoder is used to collect the robot’s real-time speed and mileage data. These data are then transmitted to the trajectory tracking node via RS485 communication. At the upper-level IPC, the environmental perception and navigation nodes are developed in C++, while the manual control node is programmed in Python 2.7.

### 3.3. Simultaneous Localization and Mapping

In complex and uncertain downhole environments, the robot perceives surroundings and adjusts self-localization via onboard sensors, leverages point cloud data and its physical model for collision detection and obstacle avoidance to support autonomous motion—aligning with the digital twin’s virtual-reality mapping concept. Advanced SLAM technology reconstructs post-disaster roadway topography for rescue guidance; the SLAM module handles low-level sensing (real-time localization, 3D point cloud mapping, pose estimation) while the digital twin undertakes high-level tasks (state visualization, human–robot interaction, rescue decision support), forming a hierarchical closed loop where SLAM provides raw data, the digital twin issues navigation commands, and SLAM feeds back updated data. Briefly illustrated via the LI-SLAM node in Figure 4, the localization and mapping module adopts a previously proposed model [26], using a tightly coupled LIDAR-IMU method to provide real-time localization for trajectory planning and precise point cloud registration for high-precision mapping, ensuring map-reality alignment and reliable data for robots in narrow alleyway-like environments.

### 3.4. Digital Model of CMRR

**Figure 5:**
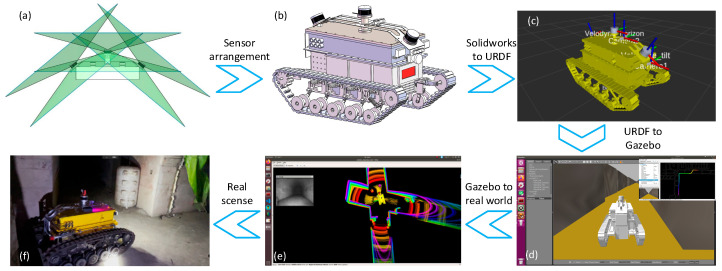
The implementation process of digital twin from modeling to real scenes. (**a**) Design of the sensor installation locations based on the LiDAR sensor’s effective area. (**b**) Design and assembly of an accurate robot model in SolidWorks 2021. (**c**) Conversion of the URDF model and determination of the positional mapping relationship from each sensor to the robot base coordinates. (**d**) Importing into Gazebo 9 to implement the physical scene simulation debugging. (**e**) Digital twin interface mapping and video surveillance windows in real scenes. (**f**) The real scene in Sub-figure (**e**).

The digital twin system is a dynamically feedback digital mirror that enables data interaction, synchronized feedback and interaction monitoring between physical and twin scenes based on device operation perceptual data. It requires a complete real-world robot with sufficient communication interfaces and data interaction rules, as well as an accurate digital model adhering to geometric, physical, behavioral and rule-based constraints to fully represent the real robot and its sensors. ROS provides an RVIZ interface for visualizing sensor data, robot self-state and environmental information, and is fully compatible with Gazebo. By exporting the robot’s URDF model via SolidWorks 2021 3D software to configure physical parameters, the robot’s structural design is driven by sensor hardware specifications to develop an accurate 3D model, which is imported via a URDF plug-in to guide physical transformation and achieve virtual-real mapping. The digital twin interface is designed using ROS reserved software interfaces, and Figure 5 shows the robot’s entire workflow from 3D modeling to field application experiments.

### 3.5. Digital Twin Interface

**Figure 6:**
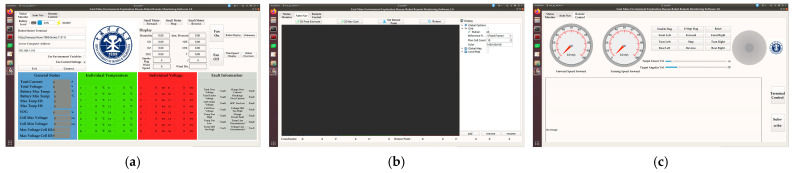
Robot digital twin interface. (**a**) Environment status display interface; (**b**) navigation control interface; (**c**) remote control and video information display interface.

For the CUMT-V(C) system, a new Qt 5-based interface under Ubuntu replaces the original Windows-dependent Visual Basic interface; ROS nodes manage its core algorithms with peer-to-peer communication via message publishing and subscription. The digital twin interface is shown in Figure 6. Additionally, after mapping physical data into a virtual form, the digital twin system tracks the physical equipment’s real-time/historical data and operational status, supported by a communication node for data interaction between the robot and the controller’s upper computer; a digital twin interface with three pages (robot status monitoring, autonomous navigation, remote control) displays the robot’s real-time status and environmental conditions.

The communication node performs the real-time keyboard remote control and cruise control of the coal mine rescue robot. At the hardware level, wireless MESH is used as a physical relay node, and the connection is established through TCP/IP. At the software level, the remote-control terminal and the robot share one ROS master. Thus, they can share ROS topics and node messages with each other and, therefore, perform remote data interaction, publishing, and receiving from the robot in real time. The digital twin system runs locally on the robot’s onboard IPC, where all sensor data is collected, processed, and integrated in real time. The remote monitoring terminal only connects to the digital twin interface node on the robot via a remote control node, and no additional raw data transmission or data processing is needed, thereby effectively reducing the occupation of communication bandwidth.

To improve rescue command efficiency, the digital twin interface adopts a three-layer display strategy. The core warning layer presents robot battery, positioning, fault and gas concentration data with red highlights for hazards. The environment perception layer displays 3D point cloud maps, real-time images and terrain rendering. The navigation control layer supports target setting, path preview and manual control. Clear visual classification reduces redundant information and enhances operational efficiency.

As shown in Figure 6a, the status monitoring page feeds back robot battery status, node information, lower computer IP and fault alerts. External environmental data, including temperature, humidity, wind speed and harmful gas concentrations, are collected by CD10 sensors. Presented in Figure 6b, the autonomous navigation page is developed based on ROS interfaces with functions similar to RVIZ. It enables target configuration, navigation preview, map loading and multi-point calibration to realize fixed-point cruise in unknown scenarios. Figure 6c illustrates the remote control page. It supports keyboard operation with real-time velocity feedback and real-time camera viewing. Manual control is enabled by default and overrides autonomous navigation. This high-priority manual intervention effectively prevents potential collision risks during robot movement.

## 4. Three-Dimensional Point Cloud-Based Path Planning Method

### 4.1. Terrain Analysis

**Figure 7:**
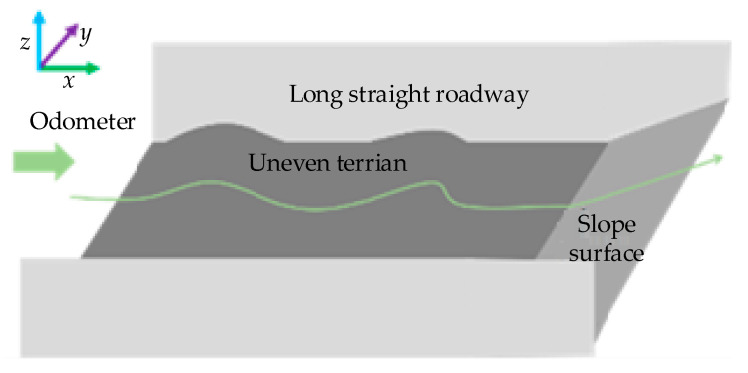
Non-flat terrain in underground environments. In this case, when directly projecting LiDAR point clouds onto a 2D plane, misjudgments about low obstacles or slopes will occur, causing the robot to be unable to pass through areas that should be passable.

Most conventional obstacle avoidance algorithms assume robots operate in a planar space. They thus project 3D LiDAR point clouds onto a 2D plane, classifying these 2D points and their adjacent expanded layers as obstacles, with ROS’s open-source Move_Base method as a typical example. However, underground terrains rarely fit an ideal plane. As shown in Figure 7, undulations, uphill, or downhill segments are common here, and these topographic features are prone to misidentification as obstacles.

**Figure 8:**
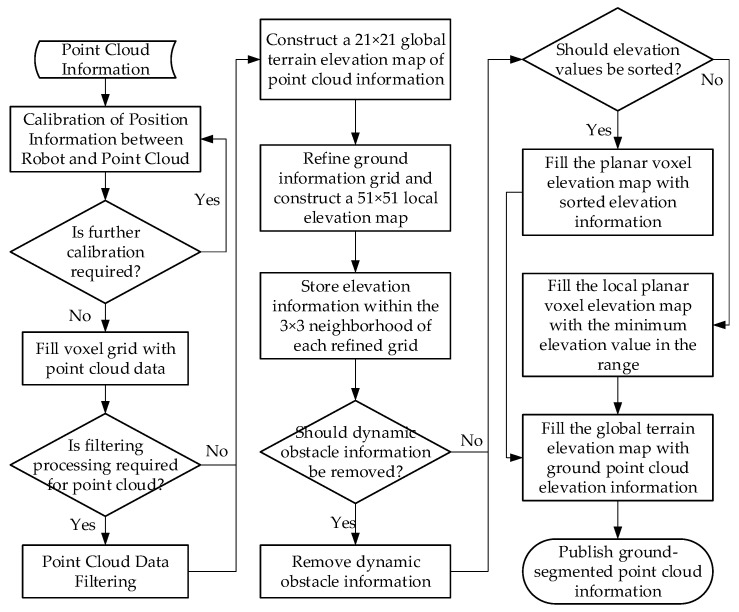
The main workflow of the terrain analysis module.

To solve this problem, this section proposed a terrain analysis method, which is similar to previous studies [49]. It constructs a cost map associated with each point on the point cloud map. The cost is determined by the degree of local terrain flatness. We do not directly use the *z*-axis height data of the point cloud acquired by LiDAR as the ground height. Instead, we calculate the local grid height of the point cloud and estimate the ground elevation by analyzing the distribution of the data points. Higher grids corresponding to protruding obstacles (e.g., tunnel walls, post-disaster collapses and falling rocks) are thus assigned higher traversal costs, thereby achieving accurate segmentation of passable areas. The main workflow of the terrain analysis module is shown in Figure 8. Terrain elevation information can be divided into two layers. The first layer is a terrain analysis area with a voxel size of 21 × 21 and a resolution of 1 m per voxel. It is mainly used to distinguish passable and impassable areas, segment feasible ground areas based on this map, and realize terrain analysis of the regional environment. The second layer is a path planning area with a voxel size of 51 × 51 and a resolution of 0.2 m per voxel, which is primarily applied to collision detection for path planning within a local scope. The specific processing methods for point cloud information are as follows.

Our method takes the LiDAR $p_{LiDAR}$ mounted on the CMRR as the center, projects the 3D point cloud onto a 2D plane, and constructs a grid map. According to the relative distance between the point cloud and the robot, the point cloud $p_{terrian}$ that meets the conditions is inserted into the corresponding 2D grid. For $p_{terrian}$, the following conditions must be satisfied:

(1) The height of the point cloud is greater than the preset minimum point cloud processing height threshold $\phi_{\min}$.
(2) The height of the point cloud is less than the preset maximum point cloud processing height threshold $\phi_{\max}$.
(3) The planar distance between the point cloud and the robot (without considering the *z*-axis distance difference) is less than the preset maximum point cloud processing distance threshold $\phi_{dis}$.

Using formulas to abstract the conditions, it can be considered that $p_{terrian}$ satisfies the formulas:

(1) $$\left\{\begin{array}{c} p_{terrain}.z>\phi_{\min}\\ p_{terrain}.z<\phi_{\max}\\ \sqrt{{\left(p_{terrain}.x-p_{LiDAR}.x\right)}^{2}+{\left(p_{terrain}.y-p_{LiDAR}.y\right)}^{2}}<\phi_{dis}. \end{array}\right.$$

Assuming the size of the grid is *voxelsize*, to balance the recording of environmental information about obstacles within a specific range around the robot and the improvement of computational efficiency, each point cloud point needs to be mapped to a corresponding grid. The formulas for calculating the grid indices of the point cloud are given as follows:

(2) $$\left\{\begin{array}{l} indX=\mathrm{int}(\frac{p_{terrain}.x-p_{LiDAR}.x}{voxelsize}+\frac{voxelsize}{2})\\ indY=\mathrm{int}(\frac{p_{terrain}.y-p_{LiDAR}.y}{voxelsize}+\frac{voxelsize}{2}) \end{array}\right..$$

**Figure 9:**
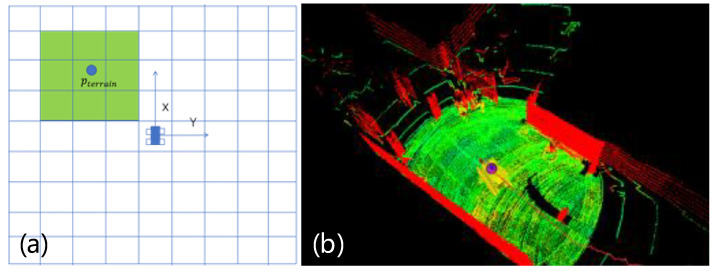
Schematic and visualization of terrain analysis. (**a**) Schematic diagram of terrain analysis. (**b**) Visualization of the terrain analysis module in Rviz. The green, red, and yellow (except for the robot model) colors, respectively, represent the level ground that can be passed, the upright flat obstacles such as walls, and the low obstacles, while the black airspace is the undetected area.

Since point $p_{terrian}$ is derived from SLAM module-output point cloud data, and the module’s single-frame point cloud count is relatively small (insufficient for accurate terrain reconstruction), each point cloud is copied to the surrounding grids during point cloud grid index calculation. For each 3 × 3 grid, the minimum *Z*-axis coordinate of all contained point clouds is adopted as the local elevation reference $z_{ref}$ for grids in that region—typically representing ground point height within the regional grids. As illustrated in Figure 9a, each point cloud’s local elevation value is determined via the surrounding 3 × 3 grids’ elevation references. For each point $p_{terrian}$ in the grids, the corresponding point cloud’s reflectivity is overwritten with the local elevation value:

(3) $$p_{terrian}.\mathrm{intensity}=p_{terrain}.z-z_{ref},$$

Figure 9b shows a topographic map of the 10 m × 10 m range in the alleyway, with the robot model in the center of the map. In addition, the terrain analysis module can handle areas of blank data points on the topographic map due to negative heights. When this module is turned on, the negative height region will be defined as the non-traversable state.

**Figure 10:**
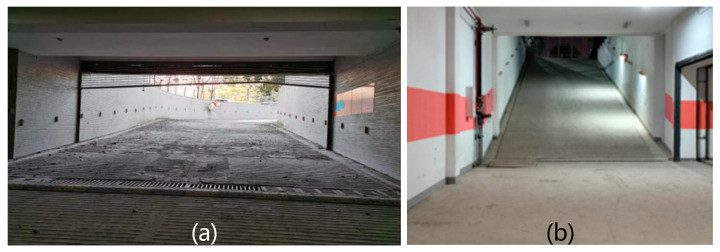
Underground long slope scenarios. The slope in (**a**) is approximately twice that in (**b**). In such an environment, the entire uphill section will be misidentified as an obstacle, resulting in navigation failure.

However, long and steep slopes, as shown in Figure 10, are common in underground environments. Traditional local ground traversability analysis performs poorly in such scenarios, especially when the slope angles are large. Significant local elevation differences exist between the slope point cloud and its surrounding point cloud, which leads to the slope being misclassified as an obstacle. To address this issue, we proposed a slope fitting strategy to process the forward point cloud, thereby ensuring the robot’s traversability in underground slope scenarios.

During CMRR point cloud data collection, the RANSAC algorithm fits a plane to the point cloud within a specific forward distance and angle range. The target fitted plane is expressed by the following equation:

(4) ax+by+cz+d=0,

where *a*, *b*, and *c* denote the normal vector components of the fitted plane (defining its orientation), and *d* is the plane offset. For plane fitting, three points are randomly sampled from the target point cloud set; the plane’s normal vector and offset are calculated using these points, followed by constructing two point-pair vectors:

(5) $$\left\{\begin{array}{l} v_{1}=(x_{2}-x_{1},y_{2}-y_{1},z_{2}-z_{1})\\ v_{2}=(x_{3}-x_{1},y_{3}-y_{1},z_{3}-z_{1}) \end{array}\right.,$$

The normal vector of the fitted plane is the cross product of the two point-pair vectors:

(6) $${\left(a,b,c\right)}^{T}=v_{1}\times v_{2}=\left|\begin{array}{ccc} i & j & k\\ x_{2}-x_{1} & y_{2}-y_{1} & z_{2}-z_{1}\\ x_{3}-x_{1} & y_{3}-y_{1} & z_{3}-x_{1} \end{array}\right|,$$

Once the plane’s normal vector is obtained, substituting one of the points into the plane equation solves for offset *d*, yielding all parameters of the current fitted plane. For each point $\left(x_{i},y_{i},z_{i}\right)$ in the set, the vertical distance from the point to this fitted plane is calculated as follows:

(7) $$e_{RANSAC}=\frac{\left|ax_{i}+by_{i}+cz_{i}+d\right|}{\sqrt{a^{2}+b^{2}+c^{2}}},$$

If the error of a point relative to the fitted plane is less than the threshold, the point is regarded as an inlier (a point lying on the plane), and the number of such inliers in the point cloud is counted. Through multiple iterations, the plane parameters corresponding to the maximum number of inliers are selected as the final fitted plane. After the plane is obtained, the inclination angle of the plane is calculated based on its parameters:

(8) $$\theta =\arctan\left(\frac{\sqrt{a^{2}+b^{2}}}{\left|c\right|}\right)\cdot \frac{180}{\pi },$$

The plane angle of the area ahead of the CMRR can be obtained. The obstacle height threshold is preset as $\phi_{obs}$. Since the reflectivity of the terrain point cloud has been overwritten as the local elevation value in the terrain analysis, it is necessary to rewrite the reflectivity value for the local elevation value of the terrain point cloud on the plane:

(9) $$p_{terrain}.\mathrm{intensity}=\left\{\begin{array}{l} p_{terrain}.\mathrm{intensity},\;(p_{terrain}.\theta \leq 10^{o})\\ \phi_{obs}+\beta (p_{terrain}.\mathrm{intensity}-\phi_{obs}),\;(10^{o}\leq p_{terrain}.\theta \leq 30^{o})\\ \phi_{obs},\;(p_{terrain}.\theta >30^{o}) \end{array}\right.,$$

where β denotes the local elevation penalty coefficient with a value range of (0, 1). After considering the CMRR’s climbing capability, a passable slope threshold is configured. Specific processing strategies are implemented based on the forward plane’s slope angle, as detailed below:

1. Slopes < 10°: These are rarely misidentified as obstacles during local elevation calculation, so the original elevation value is retained without adjustment.
2. Slopes 10°~30°: Presumed passable by default. To prevent the point cloud’s local elevation from exceeding the obstacle height threshold (due to the slope itself), the point cloud’s reflectivity is overwritten using the obstacle height threshold and current local elevation. A penalty (regulated by β) is also imposed to avoid the robot prioritizing sloped paths.
3. Slopes > 30°: The corresponding point cloud is directly marked as impassable. This eliminates navigation failure risks by preventing the robot from attempting slopes beyond its climbing capability and avoiding traversal impossibilities.

**Figure 11:**
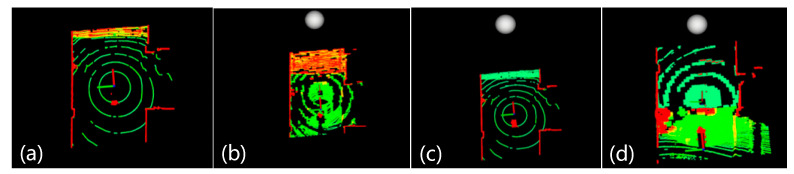
Experimental results of terrain analysis. Among them, (**a**,**b**) represent the experimental results of the benchmark algorithm, and (**c**,**d**) represent those of the algorithm proposed in this paper. The green area in the figure represents the accessible region, while the red area denotes the inaccessible region.

Figure 11 compares the proposed terrain analysis method with the benchmark algorithm, where red areas denote impassable regions calculated by the proposed method. As observed, the benchmark algorithm performs poorly in slope scenarios with natural height differences (Figure 11a,b). In contrast, the improved algorithm (Figure 11c,d) correctly identifies slope areas previously misidentified as obstacles, ensuring the robot navigates smoothly through slopes to complete the task. The proposed slope-fitting terrain analysis performs well in continuous static slopes and effectively solves the problem of slope misidentification as obstacles.

### 4.2. Global Maximum Likelihood Probability Search

**Figure 12:**
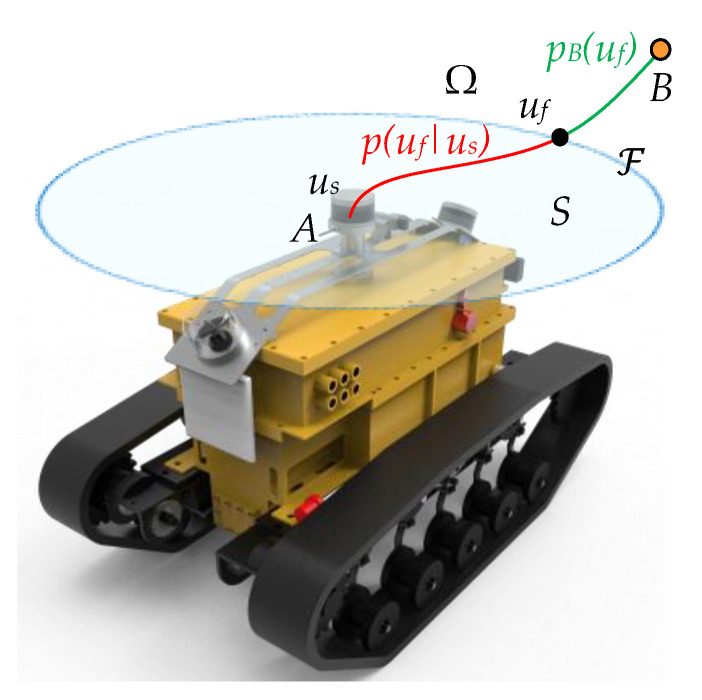
Schematic diagram of the path planning problem model. The light blue area, the dark blue curve shows the sensor range *S* and the sensor boundary F, respectively. Red curve (*u_s_* to the intersects with F at *u_f_*) and green curve (*u_f_* to *B*) form the navigation path from *A* to *B*. Entire area **Ω** as the configuration space of the robot.

The navigation problem can be constructed as a mathematical probability model as shown in Figure 12. Ω⊂R, A∈Ω, B∈Ω, and S⊂Ω, respectively, denote the robot’s motion space, the robot’s starting position, the robot’s target position, and the range of the environment that can be sensed by the laser sensor. The obstacles within the range of *S* are considered to be known with a certain probability. The probability density of the area beyond the sensor boundary F∈Ω range can be obtained from the obstacles on the a priori map. When the robot starts moving from point A, due to its physical constraints, lateral movement is impossible, so there will be no case where the planned path does not intersect with F. The initial movement state of the robot is defined as $u_{s}$. The planning problem is defined as maximizing the probability $P_{B}(u_{s})$ of reaching point *B* from the initial state ${\tilde{u}}_{s}$:

(10) $${\tilde{u}}_{s}=\arg\underset{u_{s}}{\max}P_{B}\left(u_{s}\right).$$

For a given initial state $u_{s}$, point *A* and point *B* are connected into a possible motion path by the red curve and the green curve. Define $u_{f}$ as the state of the robot when it passes through the sensor boundary F; then its conditional distribution probability density is $p(u_{f}|u_{s})$, and the probability density function of $p_{B}(u_{s})$ can be expressed as:

(11) $$p_{B}\left(u_{s}\right)=\int p_{B}\left(u_{f}\right)p\left(u_{f}|u_{s}\right)du_{f},$$

where $p_{B}(u_{s})$ denotes the probability density in the state of motion $u_{s}$ and $\left\{\xi_{i}|i=1,2,\ldots ,n;\;n\in N\right\}$ denotes a single sample $p(u_{f}|u_{s})$. Based on the Monte Carlo sampling theory for stochastic approximate inference, the above formula can be transformed into the following form:

(12) $$\int p_{B}\left(u_{f}\right)p\left(u_{f}|u_{s}\right)du_{f}\approx_{n↑\infty }\frac{1}{n}\sum_{i=1}^{n}p_{B}\left(\xi_{i}\right).$$

Treating *n* as a constant, Equation (12) can be regarded as the discretized form shown in Equation (13):

(13) $$p_{B}\left(u_{s}\right)\approx \frac{1}{n}\sum_{i=1}^{n}p_{B}\left(\xi_{i}\right).$$

The above formula indicates that the problem of the continuous sample distribution model of the probability density function $p(u_{f}|u_{s})$ for navigating from the initial state $u_{s}$ at point *A* to the terminal state $u_{f}$ at point *B* on the boundary F can be discretized by extracting *n* samples (where n≫1) from the conditional distribution $p_{B}(u_{s})$, and the optimization problem can be solved by converting a limited number of paths and voxels.

**Figure 13:**
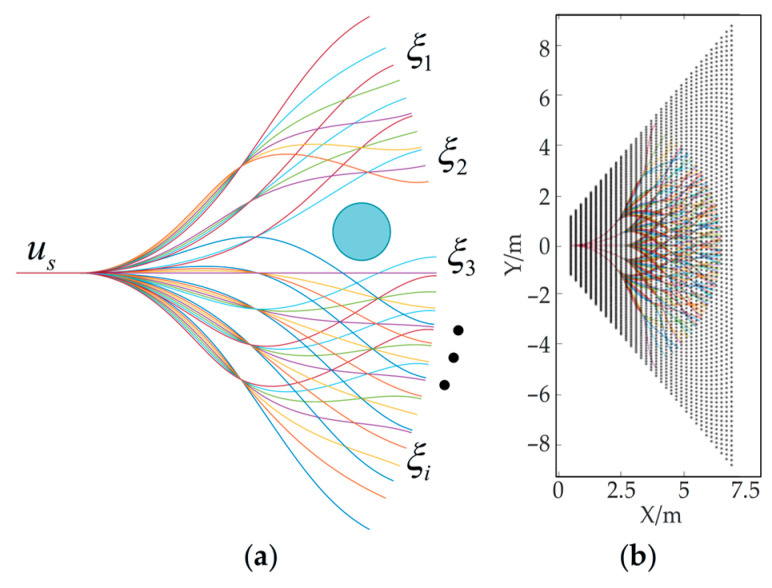
Schematic diagram of path bundle based on probabilistic occupancy. (**a**) The schematic diagram of collision detection, where the blue dot represent obstacles; (**b**) the diagram of the occlusion relationship between the offline trajectory lookup table and voxels.

To solve the problem of high computational complexity in online path planning, this paper adopts an offline preprocessing scheme: first, an offline trajectory lookup table is pre-generated, then a collision index table between voxels and occluded paths is constructed, and both files are stored in the robot’s IPC. Subsequently, collision detection can be carried out based on obstacle data obtained by sensor scanning to filter out paths occluded by obstacles, and then the optimal pre-generated path is selected online according to the cost rules to finally complete path planning. Figure 13 shows a schematic diagram of collision detection, as well as a schematic diagram of the occlusion relationship between the offline trajectory lookup table and voxels.

As shown in Figure 13, Figure 13a indicates that when some paths are occupied by obstacles, the passable probability of those paths is 0. The black dots in Figure 13b correspond to the voxels in the terrain analysis map, and the trapezoid formed by them represents the obstacle avoidance perception range of the sensor. Moreover, the closer to the robot body (origin), the higher the voxel density, indicating that the terrain detection is more refined. Since the boundary F is discretized, it can be linked to the voxel grid generated by the point cloud map, and a Boolean function $bool(\xi_{i})$ is used to represent the collision relationship. The probability of reaching the goal for all the path groups can be expressed as:

(14) $$P_{B}\left(u_{s}\right)\approx \frac{\sum_{i=1}^{n}bool\left(\xi_{i}\right)p_{B}\left(\xi_{i}\right)}{\sum_{i=1}^{n}bool\left(\xi_{i}\right)},\;\;bool\left(\xi_{i}\right)=\left\{\begin{array}{l} 1,\;\;\;\xi_{i}\;is\;unoccluded,\\ 0,\;\;\;otherwise. \end{array}\right..$$

Afterward, the probabilities $P_{B}(u_{s})$ of all the path groups are calculated online, and the path group having the highest $P_{B}(u_{s})$ is considered as the robot’s execution path. The probability transfer rule $p_{B}(\xi_{i})$ is calculated in agreement with Falco [36].

### 4.3. Offline Bézier Curve Trajectory Generation

In this study, the Bézier curve, a special case of B-spline curves, is adopted to generate the trajectory lookup table for local planning instead of monomial basis polynomials. Proposed by Pierre Bézier, this vector curve is widely applied in engineering drawing, animation design and other fields, and has also been extensively used in the path planning of robots and UAVs owing to its excellent inherent properties: it passes through the start and end control points but never the intermediate ones, maintains tangential connection with the guide lines of the initial and terminal segments, is always confined to the convex hull formed by its control points, and features a recursive nature where the *n*th-order derivatives of an *n*-1st-order Bézier curve are still Bézier curves.

It can then be deduced from these properties that a Bézier curve with an order greater than 5 should have 4th-order derivatives. Therefore, it is very suitable for the optimization of the control quantities (velocity, acceleration, jerk, and snap) of the robot. The general *n*th-order Bézier curve segment is expressed as:

(15) $$B_{j}(t)=c_{j}^{0}b_{n}^{0}(t)+c_{j}^{1}b_{n}^{1}(t)+\ldots +c_{j}^{n}b_{n}^{n}(t)=\sum_{i=0}^{n}c_{j}^{i}b_{n}^{i}(t),t\in [0,1],$$

where $b_{n}^{i}(t)$ is the *i*th-order Bernstein polynomial basis defined as:

(16) $$b_{n}^{i}(t)=C_{n}^{i}t^{i}{\left(1-t\right)}^{n-i}=\frac{n!}{i!(n-i)!}t^{i}{\left(1-t\right)}^{n-i},i=0,1,2,\ldots ,n,$$

where $c_{j}=[c_{j}^{0},c_{j}^{1},\ldots ,c_{j}^{n}]$ denotes the set of control points of the *j*-th segment of the Bézier curve and C is the Combination symbol.

Since Bézier curves lack intermediate control points, an approach analogous to that of quasi-uniform B-splines is employed to generate Bézier curves more suitable for the proposed local planning task. Specifically, a small variation parameter *τ* is defined, enabling the Bézier curve to incorporate constraint points around intermediate control points (*key*). Except for the head and tail control points of the curve, the number of these additional constraint points is regulated by a constraint point vector w:

(17) $$\left\{\begin{array}{l} B_{j}(t)=w\sum_{i=0}^{n}c_{j}^{i}b_{n}^{i}(t),t\in [0,1]\\ w={\left[w_{1},\ldots ,w_{key-1},w_{key},w_{key+1},\ldots ,w_{n}\right]}^{T},w\in \{0,1\}\\ c_{j}^{key-1}-c_{j}^{key}=c_{j}^{key}-c_{j}^{key+1}=\tau ,\;\;\;0<key\leq n \end{array}\right..$$

**Figure 14:**
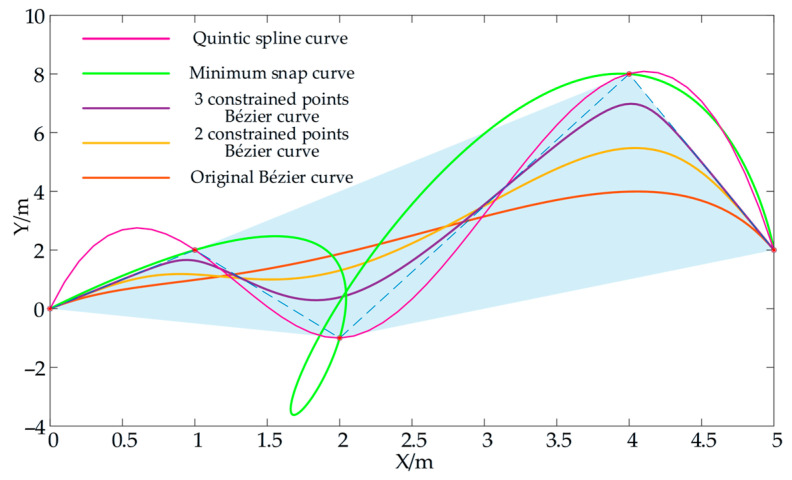
Comparison of spline curve and Bézier curve trajectories. The magenta, green, red, orange, and purple lines show the minimum snap curves, the quintic spline curve, the original Bézier curve (O-BC), the Bézier curve after adding two constraint points near each intermediate control point (2C-BC), and the Bézier curve after adding three constraint points near each intermediate control point (3C-BC), respectively. The dashed line is the guiding line of the curve, while the blue area is the convex cell enclosed by four control points.

Figure 14 illustrates the difference between the spline curves, minimum snap curves and Bézier curves in path planning applications. Since the spline curve and minimum snap curves should pass through the control points, the optimized trajectory is generated with a very inappropriate trajectory in the middle (between control points 2 and 3), while it is ensured that the Bézier curve is always within the convex cell formed by the control points. In addition, the Bézier curve can be controlled close to the middle control point by increasing the number of constraint points around it. Moreover, the generated curve is still continuous and derivable everywhere.

**Figure 15:**
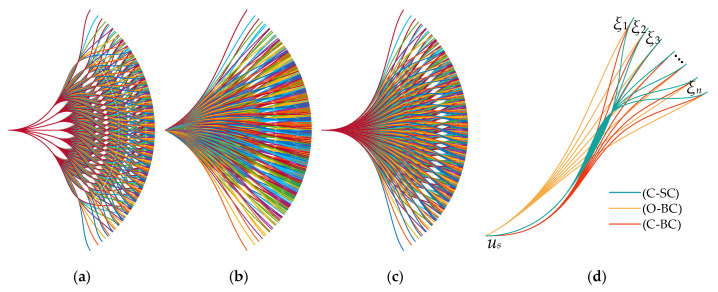
Offline lookup table for local trajectory. (**a**) Cubic spline curve (C-SC). (**b**) Original Bézier curve (O-BC). (**c**) Bézier curve with constraint points (C-BC). (**d**) Comparison of the curve shapes of the three path generation methods.

Graphs of spline curves and Bézier curves generated based on the aforementioned rules are presented in Figure 15. Falco [36] adopted cubic spline curves as the benchmark for generating offline path lookup tables, as shown in Figure 15a. Since the probability from the starting point to the target point can be approximated by sampling the sensor boundary *F*, we set the paths to be distributed in three sections, and the starting point of each section must pass through a control point. Therefore, the paths from the starting point A to the boundary *F* are sampled along seven directions, with the angle range of the seven directions being from −27° to 27° and the angle interval between adjacent directions being 9°. The paths generated along one direction form a group, resulting in a total of seven groups of paths. Each group of paths is further sampled into sub-branches along seven directions, and each sub-branch is provided with seven evenly distributed endpoints reaching the sensor boundary F; that is, each group has 7 × 7 = 49 sub-branch paths, and seven groups of paths have a total of 7 × 7 × 7 = 343 paths. Each path is generated according to the kinematic constraints of the vehicle and is regarded as a feasible path from the starting point $u_{s}$ to the sensor boundary *F*. Figure 15a shows the paths generated by sampling along seven directions and the overall path diagram, and the state of the end paths can be regarded as Monte Carlo samples $\left\{\xi_{i}|i=1,2,\ldots ,n;\;n\in N\right\}$ for $p(u_{f}|u_{s})$ sampling. Each path in the offline trajectory library is generated one by one through MATLAB 2016b curve functions with preset sampling points. All trajectories are saved in *.ply* format and stored on the robot IPC, together with collision detection files in *.txt* format.

**Figure 16:**
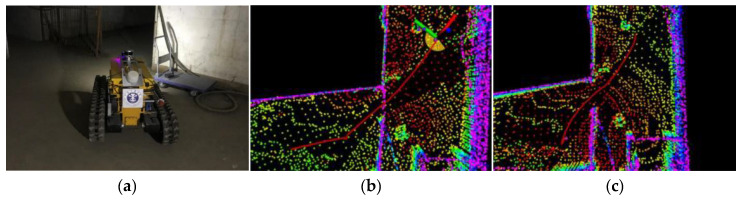
The situation where different trajectories pass a right-angle bend. (**a**) On-site photograph of the turning experiment; (**b**) travel trajectory generated by the O-BC trajectory; (**c**) travel trajectory generated by the C-BC trajectory.

Figure 15b displays the trajectory diagram generated by a general Bézier curve. With all control points unchanged, all paths are distributed within the convex hull enclosed by these control points. Furthermore, due to the inherent characteristics of the general Bézier curve, the paths exhibit almost no bends, which is highly unfavorable for obstacle avoidance, as can be seen in Figure 16b. Figure 15c shows the path set generated in accordance with the rules outlined in Equation (17), where $c=[c^{0},c^{key_{1}-1},c^{key_{1}},c^{key_{1}+1},c^{key_{2}-1},c^{key_{2}},c^{key_{2}-1},c^{3}]$ and ***w*** = [1,1,1,1,1,1,1,1]^T^. Specifically, constraint points are added around the intermediate control points (excluding the start and end points). It can be seen that the trajectories can be brought closer to the control points, and the paths exhibit significant curvature. Compared with the paths in Figure 15a, however, these paths are more uniformly distributed within a finite area, providing greater flexibility for path selection. Figure 15d presents a comparison of the three curve branch bundles. Notably, the positions of the start and end points remain constant regardless of the curve shape.

The turning performance of robots is also a key research focus in path planning. Although turning only accounts for a small part of the overall driving trajectory, it largely determines the performance of path planning algorithms. As shown in Figure 16b, the original Bézier curve shows no obvious turning tendency and travels nearly in a straight line when passing through the iron gate. In contrast, the Bézier curve with key points in Figure 16c delivers distinct obstacle avoidance movements. It demonstrates that the Bézier curve integrated with key points is more conducive to robot turning and obstacle avoidance.

### 4.4. Optimal Trajectory Selection Function

The baseline algorithm [36] selects the optimal path using only an empirical function, typically based on minimum cost, thus preferring wall-adjacent paths when turning. However, this method is unsuitable for robots with relatively large dimensions, as it easily causes collisions. To keep navigation paths away from walls, a combined function is designed, integrating the original objective function and a Voronoi diagram-based constraint function:

(18) $$J=J_{ori}+J_{vor}.$$

The original objective function is given by:

(19) $$J_{ori}=w_{ori}(1-\sqrt[{4}]{w_{dir}\cdot \Delta \varphi_{diff}})\cdot \Delta {\varphi_{rot}}^{4},$$

where $\Delta \varphi_{diff}$ is the angular difference between the path and the target point, $\Delta \varphi_{rot}$ represents the angular difference between the direction of the path and the current robot orientation, *w_ori_* is the function cost term, and *w_dir_* is the path direction weight.

The Voronoi term (*J_vor_*) guiding the path away from the obstacle through the nodes in the Voronoi field:

(20) $$J_{vor}=w_{vor}\left(\frac{\alpha }{\alpha +d_{obs}(x,y)}\right)\left(\frac{d_{vor}(x,y)}{d_{obs}(x,y)+d_{vor}(x,y)}\right)\left(\frac{{\left(d_{O}-d_{vor}^{\max}\right)}^{2}}{{\left(d_{vor}^{\max}\right)}^{2}}\right),$$

where *w_vor_* represents the weight of the Voronoi term, *d_obs_* is the distance to the nearest obstacle, *d_voi_* is the positive distance to the nearest edge of the Generalized Voronoi Diagram (GVD), $d_{vor}^{\max}$ denotes the maximum distance from the edge of the Voronoi diagram, α>0 and $d_{O}$ are, respectively, the constants that control the decay rate and the maximum effective range of the obstacle distance in the environment, and $J_{vor}=0$ when $d_{O}>d_{vor}^{\max}$. Note that in practice, the Voronoi map raster resolution is consistent with the voxel grid map generated by the terrain analysis, which can greatly reduce the computational load of the system.

**Figure 17:**
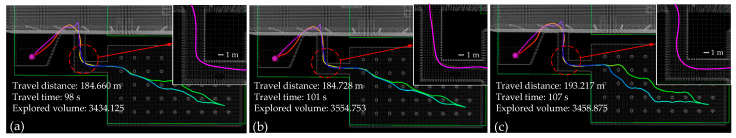
Full process diagram and local inflection trajectory diagram in the simulation experiment. (**a**) The path diagram using C-SC offline lookup table and the *J_or__i_* cost term. (**b**) The path diagram using C-BC offline lookup and the *J_or__i_* cost term. (**c**) The path diagram using C-BC offline lookup and the *J_or__i_* + *J_vor_* cost term, which is fitted with a purple line because the two paths are approximated back and forth. The gradient-colored lines indicate the travel path of the robot.

Figure 17 shows the path planning effects under three modes. Figure 17a presents the path diagram of the C-SC + *J_ori_* scheme. From the perspective of the overall travel trajectory, the C-BC + *J_ori_* scheme (Figure 17b) tends to follow the already traversed path when returning, with almost no increase in the total path length and exploration time, and also tends to move away first when passing through right-angle bends. It can be seen from Figure 17c that the planning of the C-BC + *J_ori_* + *J_vor_* scheme tends to select paths far away from obstacles; therefore, the total path length is increased by 4.6% compared with the C-BC + *J_ori_* scheme, the total exploration time is also increased by 9.2% accordingly, the exploration volume has no significant change, and the robot basically passes directly between the two pillars. The path of the C-BC + *J_ori_* + *J_vor_* scheme can keep away from right-angle wall corners and tends to move away from the wall when entering right-angle bends, maintaining a distance of more than 1 m from the wall. In contrast, the paths of the two schemes using only *J_ori_* tend to be planned close to the wall.

## 5. Field Experiments

**Figure 18:**
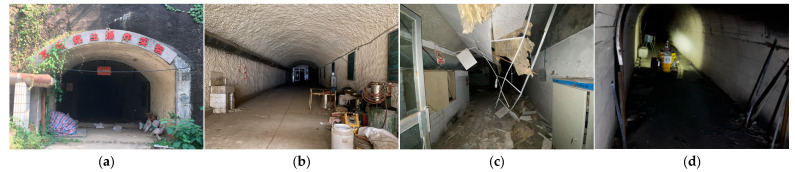
Field experiment environment. (**a**) Main entrance (Gas and Coal Dust Explosion Laboratory). (**b**) Entrance promenade. (**c**) Collapsed area. (**d**) Branch alleyway.

Field experiments were conducted in a tunnel environment within the Coal Dust and Gas Explosion Laboratory of China University of Mining and Technology. This laboratory features working conditions, including structured tiled roadways and cement-sprayed coal mine roadways. In the branch roadways, coal dust and debris (e.g., broken tables and chairs) were placed to simulate explosion scenarios; additionally, collapse traces were present at the entrances of the tiled roadways. This setup is consistent with the actual post-disaster environment of coal mines. The on-site environment of the roadways is shown in Figure 18.

### 5.1. Control Accuracy Experiments

**Figure 19:**
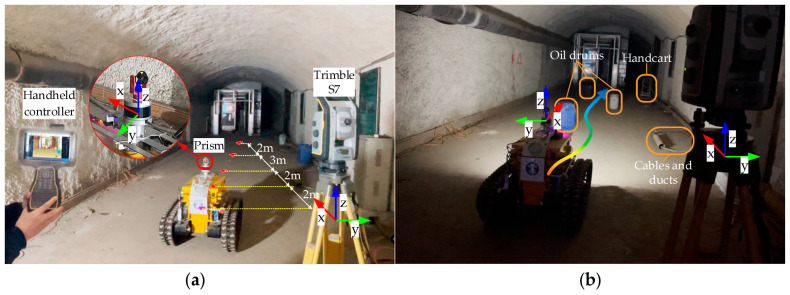
Static obstacle avoidance experiment scene. (**a**) Trimble S7 field setup approach. (**b**) Site placement program for obstacles.

To obtain the robot’s actual 3D position, a Trimble S7 total station (© Trimble Inc., Hong Kong, China) paired with a Trimble TSC7 handheld controller (© Trimble Inc., Hong Kong, China) was used to record reference values. The experimental site layout is shown in Figure 19. The Trimble S7 was operated in angular tracking mode (sampling frequency: 2.5 Hz) for auto-lock tracking. A prism was mounted on top of the robot’s LiDAR, with a default height of 0.85 m.

The lower right corner of Figure 19 indicates the global coordinate system orientation of the total station: the x-direction points parallel to the tunnel wall toward the tunnel interior, and the y-direction points rightward. The LiDAR coordinates in Figure 19a correspond to the coordinate system established by the robot’s SLAM module, where the *x*-axis points forward. Notably, the *y*-axis direction of the total station is opposite to that of the robot, as no global coordinate alignment was performed.

#### 5.1.1. Straight-Line Fixed-Point Parking Experiment

The field experiment first evaluated the robot’s linear control performance. In Figure 19a, purple pentagons denote the start and end positions, while red pentagons represent four preset stop points—resulting in a total of seven stops, each lasting 5 s. The spacing between target points is shown in the figure, with the linear test spanning a total length of 9 m. The target arrival threshold was set to 0.1 m; specifically, the robot was deemed to meet the parking condition when it entered the 0.1 m radius range of a stop point. The robot’s maximum linear speed and maximum angular speed were configured to 0.2 m/s and 0.5 rad/s, respectively.

**Figure 20:**
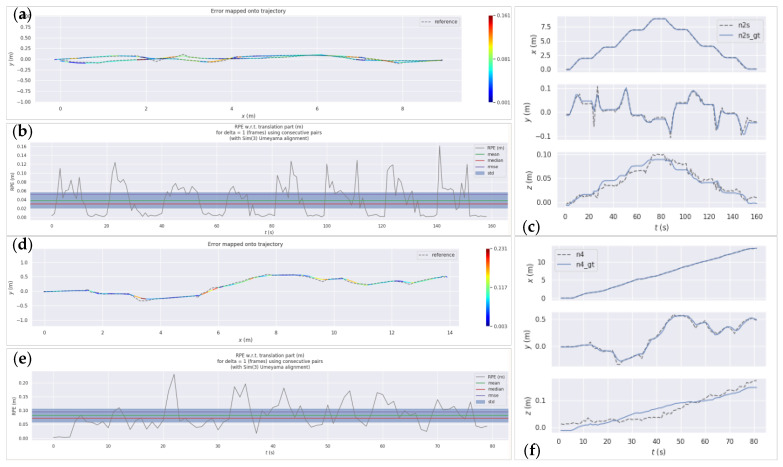
Experimental EVO evaluation chart of the control accuracy. (**a**–**c**) Straight-line fixed-point parking experiments, including two paths with back and forth. (**d**–**f**) Pile wrapping experiment. (**a**,**d**) are plots of the robot trajectory versus the true value. (**b**,**e**) are plots of the robot control accuracy evaluation. (**c**,**f**) are the values of the robot in the three directions over time.

Figure 20 presents the actual travel trajectory measured by the total station and the radar odometer trajectory generated during planning. Since no global coordinate alignment was performed, the EVO toolbox (https://github.com/MichaelGrupp/evo (accessed on 6 March 2026)) was used to evaluate relative accuracy. Additionally, the control accuracy of the navigation system was assessed based on the relative error of stop points, incorporating positioning accuracy. A comparison between the set values of the set values and the actual values measured by the Trimble S7 base station in the straight-line fixed-point parking experiment is presented in Table 2.

As shown in Table 2, the maximum positioning error at key navigational points relative to the targets is 0.088 m, the minimum error is 0.028 m, and the average error is 0.05975 m. These results demonstrate that the CUMT-V(C) robot exhibits favorable motion control accuracy. Figure 20a–c present the quantitative evaluation results of the fixed-point parking experiment. In Figure 20a, the robot exhibited nearly zero error in the x-direction, while the maximum error in the y-direction reached 0.161 m. Under MPC control, its planned total travel distance was 18.226 m, with an actual travel distance of 18.041 m, resulting in an overall deviation of 1%, and specific quantitative metrics are detailed in Figure 20b. The Relative Pose Error (RPE) curve shows eight dips, corresponding to seven stops at the four fixed stop positions, where the maximum relative deviation was less than 0.02 m. In terms of positioning accuracy, the robot’s overall mean positioning error was 0.038 m (variance: 0.44); the maximum deviation during travel was 0.161 m, with a standard deviation of 0.036 and a root mean square error (RMSE) of 0.053.

#### 5.1.2. Pile-Wrapping Experiment

The site layout of the pile-wrapping experiment is shown in Figure 19b. Three low oil drums were arranged in the tunnel as obstacles (positioned below the robot’s sensors), and a thick cable with a sleeve was placed on the ground to the robot’s left. Low illumination is the most common environment following an underground disaster; thus, this experiment was conducted at night without ambient light sources, relying solely on the explosion-proof mining lamp mounted on the robot to illuminate its front area. Notably, positioning accuracy was ensured because the laser (sensor) and total station are unaffected by illumination conditions.

Figure 20d–f present the quantitative evaluation results of the pile-wrapping experiment. Figure 20d shows an S-shaped trajectory, representing the robot’s obstacle avoidance behavior under low illumination conditions, with a maximum motion deviation of 0.231 m. The robot’s planned total travel distance was 14.466 m, while the actual travel distance was 14.232 m, resulting in an overall deviation of 1.6%.

Figure 20e illustrates the specific trend of Relative Pose Error (RPE), where the maximum deviation of 0.231 m occurred when the robot rotated to avoid the first obstacle. The trajectory’s overall mean error was 0.082 m, with a variance of 0.72, a standard deviation of 0.048, and a root mean square error (RMSE) of 0.095.

Figure 20f displays the robot’s position values in three directions over time during the experiment. The overall error in the x-direction was small, while errors in the y- and z-directions were larger, attributed to fluctuating errors of the SLAM algorithm caused by the robot’s continuous rotation and adjustment.

### 5.2. Narrow Path Crossing Experiment

**Figure 21:**
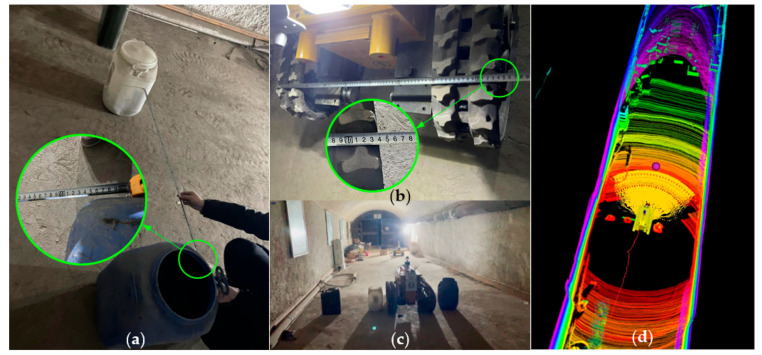
Narrow-track traversal field experiment. (**a**) Narrow path distance measurement. (**b**) Robot track width measurement. (**c**) The robot traverses the narrow path. (**d**) Map of the robot’s environment while traversing the narrow path in the navigation interface.

The narrow path crossing experiment was conducted to verify the ultimate passing performance of the robot navigation system. Figure 21 presents the experimental site layout and navigation results.

Three oil drums were placed as obstacles in the straight alleyway; among them, the drum adjacent to the wall (the black drum at the top of Figure 21a) was used to restrict free space, preventing the robot from prioritizing wider areas for passage based on its path selection strategy. Given the robot’s rectangular body, a default expanded collision margin of 0.1 m was set in the algorithm to avoid collisions with obstacles. The spacing between the white and blue oil drums and the corresponding experimental results are detailed in Table 3.

Experimental results indicate that when the obstacle spacing is 1.1 m, the narrow path provides a limited free width of 0.26 m for the robot (with a unilateral clearance of 0.13 m), and the robot can pass successfully. In contrast, narrower spacing (smaller than 1.1 m) is insufficient for the robot to traverse safely.

Figure 21c shows the robot navigating the 1.1 m wide narrow path; it can be observed that the robot passes through the middle of the path without contacting obstacles. As presented in Figure 21d, the point cloud map generated by the mapping algorithm accurately depicts the positions of the three oil drums. The red line behind the robot represents the travel trajectory recorded by the SLAM algorithm’s odometer, while the yellow free path ahead indicates available movement options. Notably, the optimal path selection function effectively identifies the optimal path, enabling the robot to avoid obstacles successfully.

### 5.3. Experiment on Dynamic Obstacle Avoidance

**Figure 22:**
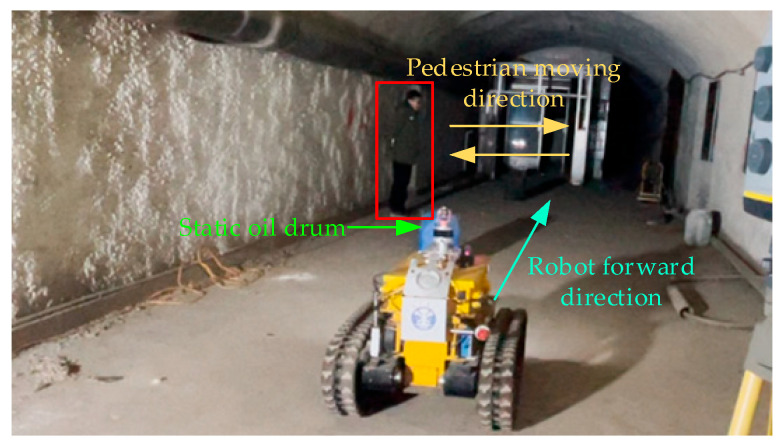
Dynamic obstacle avoidance experiment site.

Figure 22 shows the schematic diagram of the obstacle avoidance test site in a dynamic environment. An oil drum is placed in the middle of the simulated roadway as a static obstacle. A pedestrian starts moving toward the middle of the roadway after the robot passes the oil drum, and stands still upon reaching the middle of the roadway. When the robot passes through smoothly, the pedestrian returns to the original position. This test scenario is designed to test the effectiveness of the autonomous obstacle avoidance algorithm in coping with dynamic obstacles.

**Figure 23:**
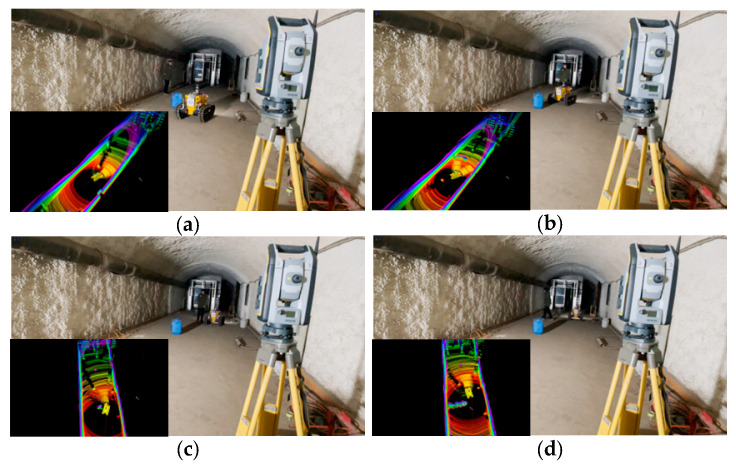
Dynamic obstacle avoidance test results and navigation interface display. (**a**) Avoiding static obstacles. (**b**) Starting to avoid dynamic obstacles. (**c**) Avoiding dynamic obstacles. (**d**) Dynamic obstacle avoidance completed.

Figure 23 presents the actual scene of the obstacle avoidance test in a dynamic environment and the display of the navigation interface. It can be seen from Figure 23a that the robot can easily avoid the static obstacle while maintaining a relatively safe distance. Figure 23b shows that during the robot’s forward movement, the pedestrian has moved to the middle of the roadway, and the navigation interface indicates that the robot has eliminated the paths in the non-free area corresponding to the pedestrian’s position. Figure 23c illustrates that the robot is avoiding the stationary pedestrian without colliding with the pedestrian during movement. Figure 23d displays the state when the pedestrian returns to the original position after the robot completes obstacle avoidance, and the point cloud information left by the pedestrian’s movement can be observed on the navigation interface.

### 5.4. Crossing the Barrier Area

**Figure 24:**
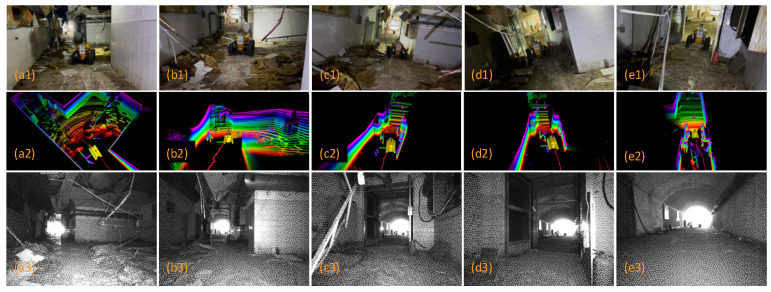
Traversing the collapse area. From top to bottom: the current area of the third view of the real shooting scene, the digital twin navigation interface, and the Realsense D435i infrared image. (**a1**–**a3**) The robot avoids collapsed obstacles; (**b1**–**b3**) The robot travels through the ruins; (**c1**–**c3**) The robot continues to avoid collapsed obstacles; (**d1**–**d3**) The robot traverses a narrow collapsed space; (**e1**–**e3**) The robot passes through a doorway. The navigation interface includes a dense point cloud of the environment (the color changes represent the height), collision-free paths (yellow sector), and a robot model (yellow color).

Figure 24 illustrates the full autonomous navigation process of the coal mine rescue robot in a simulated tunnel collapsed area. During navigation, the robot can identify and execute a collision-free path. As shown in Figure 24(a1), when crossing the flexible insulating mat, the robot can move forward steadily even with its large weight by virtue of the trapezoidal tracked structure. The robot always maintains a good grip in this scenario without obvious slippage. The influence of debris and dust on the autonomous locomotion of the robot is slight, which is an ability that ordinary wheeled robots do not possess. The robot can also traverse certain hard, collapsed objects (Figure 24(c3)) without damage, as its explosion-proof housing provides effective protection for internal components.

From Figure 24(a2), the digital twin interface clearly displays obstacles in the surrounding environment, including collapsed ceiling structures on the left and right sides ahead of the robot, and ground debris such as insulation foam. Additionally, the binocular camera’s infrared imaging function allows real-time observation of the actual environment in front of the robot, which can serve as a reference and basis for comparison.

### 5.5. Global Exploration Experiment

Global exploration was conducted in the collapsed area of the simulated roadway and the coal dust explosion experimental area. The operator clicked waypoints on the navigation interface, with the robot’s maximum linear speed and maximum angular speed set to 0.2 m/s and 0.5 rad/s, respectively. No priori maps were used during exploration; the entire test was carried out in an unknown environment without GPS availability, and positioning relied solely on the odometer fused with LiDAR and IMU.

**Figure 25:**
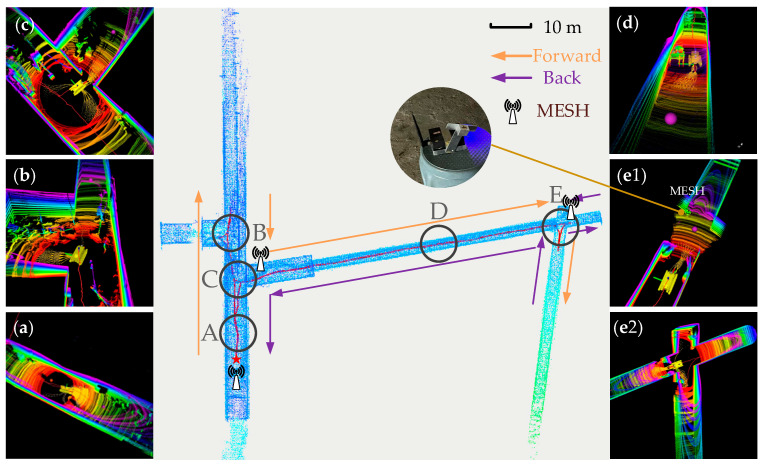
The point cloud map during global exploration and the display of the navigation interface for key scenes. (**a**) Navigation interface when circumventing road pile obstacles. (**b**) Navigation interface when the robot traverses a collapsed area. (**c**) Robot traversing a fenced area. (**d**) Robot avoiding debris in a long alleyway. (**e1**) Robot about to enter an intersection. (**e2**) Robot backing up, turning around, and then returning. The black capital letters represent the corresponding position of each subfigure in the entire exploration area.

Since wireless signals are absorbed by tunnel walls when passing through bends, wireless MESH nodes were pre-deployed at several key inflection points inside the tunnel to ensure stable communication (an example is shown in Figure 25(e1)). The robot is equipped with a mobile MESH node, enabling it to dynamically establish a high-speed communication network during online travel—thus ensuring real-time transmission of environmental data, video, and point cloud maps.

**Figure 26:**
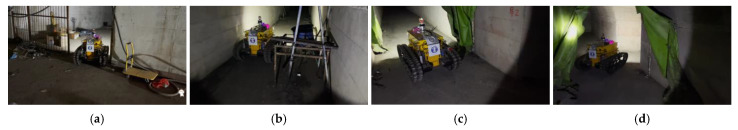
CMRR in the real scene. (**a**) The real position of the robot when passing through area C in Figure 25. (**b**) The real position of the robot when passing through area D (return) in Figure 25. (**c**,**d**) The movements of the robot when passing through area E in Figure 25 and exploring the right turn forward, reversing, and turning around to return, respectively.

Figure 25 illustrates the full exploration process in the alleyway environment and the navigation interface for key scenarios. The red line represents the robot’s navigation trajectory, the red pentagram denotes the start and end points, and the two-color arrows indicate the forward and return directions during exploration, respectively. Figure 26 corresponds to the real-world environments of several key scenarios shown in Figure 25.

In this experiment, the robot traveled a total distance of 283.556 m. During forward exploration in Area B, the robot adopted a leftward detour path to avoid obstacles and a reverse-turnaround path for return; the same reverse-turnaround-return actions were executed in Area E. Overall, the robot maintained travel near the midline in long alleyways and successfully identified collision-free paths through narrow passages and obstacle zones.

A comparison between the point cloud map from the navigation interface (Figure 25) and the real scenes (Figure 26) clearly demonstrates that the point cloud map accurately reproduces the robot’s surrounding real environment. This further verifies the effectiveness of the digital twin system designed for coal mine search and rescue robots.

### 5.6. Long-Distance Target Point Navigation Experiment

**Figure 27:**
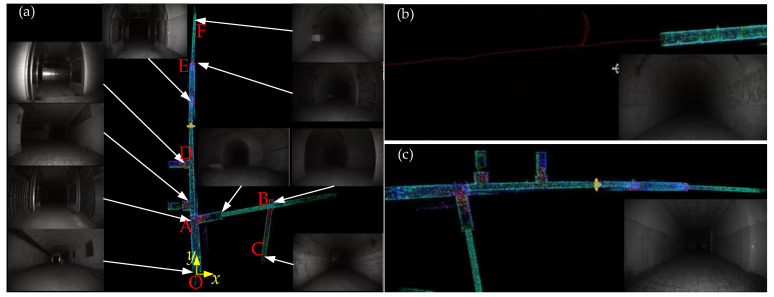
The long-distance waypoint navigation experiment of simulated tunnel. (**a**) Navigation results and key area display. (**b**) Laser point clouds degenerate. (**c**) The 3D point cloud map is restored after degradation. The red capital letters represent the key parking positions and the corresponding location of each subfigure in the entire exploration area.

To verify the stability of the navigation system, a long-distance waypoint navigation experiment was conducted in a simulated tunnel, as illustrated in Figure 27. Prior to the experiment, the collapse and obstacles in Area A (shown in Figure 25) were cleared. A total of 6 global waypoints (a–f) were set, with the stop position of each waypoint detailed in Figure 27a. All navigation parameters of the robot remained unchanged, and the map used was the prior map constructed by the robot itself.

The robot departed from the home point O (0, 0, 1.1) at the entrance of the main tunnel, navigated to waypoint A (the first fork) and dwelled there for 15 s. Subsequently, it navigated right to waypoint B (the crossroads), with no manual intervention throughout this segment. After passing the crossroads, the robot reached waypoint C—located at the end of a 5° downhill segment near an iron gate—and stayed for 15 s before returning. It made stops at waypoints B and A successively, then turned right to re-enter the main tunnel, moved forward through the vestibule, traveled straight past the first chamber, continued onward, and finally turned left to reach waypoint D (the second chamber). Following a 15-s stop at Chamber D, the robot returned to the main tunnel, continued along the main tunnel to waypoint E (the narrowed section of the tunnel), dwelled for 15 s, and then proceeded straight to waypoint F (the conveyor belt location). Due to map degradation in this tunnel segment, no additional waypoints were deployed. Ultimately, the robot returned directly to home point O, with no manual intervention during the return journey.

During the waypoint navigation experiment, when the robot traveled through the narrow tunnel where the conveyor belt is situated, a path-map mismatch occurred due to map degradation, as presented in Figure 27b. The deviated segment in the path corresponds to the robot’s trajectory when exploring and exiting the second chamber (D). As the robot continued moving into the tile-lined corridor, the path realigned with the map. Figure 27c depicts the recovery process of the 3D point cloud map after degradation. Upon returning to the home point O, the robot’s coordinates were approximately (0.5, 1.1, 1.1). Compared with its initial position, the positional deviation was roughly equivalent to the length of one robot body.

## 6. Conclusions

Coal mine intelligence has emerged as a key trend in coal science, particularly regarding autonomous navigation and digital twin technologies. The transfer of advanced surface technologies to underground coal mine applications is crucial for realizing minimal-human and unmanned coal mines. This is especially valuable in post-disaster coal mine scenarios: a fully autonomous robot—capable of making navigation decisions without human supervision or remote operation and synchronizing environmental data in real time—would be of great practical significance. As an integral component of the CMRR capability framework, we developed the CUMT-V(C), which incorporates intelligent and real-time visualization to establish a digital twin-based hardware-software system for CMRRs.

This paper proposes an offline lookup table method based on Bézier curves: compared with spline-based trajectories, it achieves flexible obstacle avoidance by efficiently sampling traversable areas via more uniformly distributed navigation trajectories. To adapt to coal mine tunnel planning, a terrain analysis method using slope fitting is introduced, endowing the CMRR with autonomous obstacle avoidance on steep terrains. Incorporating a Voronoi cost term into the optimal path cost function further enables the robot to avoid static obstacles (e.g., tunnel walls). Additionally, a visual interface allows real-time monitoring of the robot’s status by rescue personnel, with visualized data supporting both autonomous obstacle avoidance and pre-entry situational awareness. Point parking experiments and motion trajectory tests verified the navigation system’s control accuracy, while tunnel and disaster site experiments demonstrated the robot’s ability to navigate and explore unknown tunnel environments, as well as select safe paths for long-distance travel across complex terrains. The CUMT-V(C) breaks the limitation of wired control and enables the robot to move more freely in confined post-disaster spaces. It no longer needs to avoid damaging the unrecoverable towed optical fiber laid on the ground, which greatly reduces the operational pressure on rescue operators and improves safety and flexibility in disaster scenarios.

However, some practical issues were not fully considered. For instance, the communication modules were pre-deployed in field experiments. Thus, future work will add a communication node release structure to the robot’s tail to enhance the autonomous deployment capability of communication modules. Meanwhile, the terrain analysis model has limitations in extremely unstructured post-disaster scenarios, such as instantaneous collapses and dynamic falling rocks, and cannot detect these real-time terrain deformations, which may lead to system performance degradation or even failure. In addition, long-term navigation in textureless and structurally similar roadways may result in map degradation and localization drift, further affecting system stability. To mitigate these problems, we plan to introduce LiDAR temporal difference detection and adaptive slope threshold adjustment to identify dynamic terrain changes. At present, there are still 3 CUMT-V(B) robots that have not been effectively utilized. Future work will leverage these robots to conduct in-depth research on heterogeneous swarm collaborative exploration, embodied decision-making, and autonomous interaction, thereby enabling large language model-based embodied navigation for CMRRs.

## Figures and Tables

**Table 1:** The parameter comparison between the CUMT-V(C) and CUMT-V(B).

Parameters	CUMT-V(C)	CUMT-V(B)
Robot dimensions	1.24 m (L) × 0.84 m (W) × 0.8 m (H)	1.24 m (L) × 0.84 m (W) × 0.58 m (H)
Maximum walking distance	≈5 km	7 km
Maximum speed	1 m/s	1.3 m/s
Maximum climbing angle	≈25°	30°
Maximum communication distance	200 m (wireless)	50 m (wireless)/1000 m (optical fiber)
Highest obstacle height	≈150 mm (Figure 3 shows)	200 mm
Overall self-weight	≈420 kg	≈350 kg

**Table 2:** Comparison between set values and actual values of fixed-point parking experiment.

No.	Set Value (m)	Actual Value (m)	Arrival Threshold (m)	Actual Distance (m)
1	(2.0, 0, 0)	(1.935, 0.035, 0.853)	Φ0.1	0.065
2	(4.0, 0, 0)	(3.912, 0.005, 0.884)	Φ0.1	0.088
3	(7.0, 0, 0)	(6.935, −0.013, 0.916)	Φ0.1	0.065
4	(9.0, 0, 0)	(8.930, −0.023, 0.945)	Φ0.1	0.070
5	(7.0, 0, 0)	(7.028, 0.040, 0.930)	Φ0.1	0.028
6	(4.0, 0, 0)	(4.074, 0.030, 0.896)	Φ0.1	0.074
7	(2.0, 0, 0)	(2.062, −0.003, 0.875)	Φ0.1	0.062
8	(0.0, 0, 0)	(0.026, −0.042, 0.857)	Φ0.1	0.026
Average Error	-	-	-	0.05975

**Table 3:** Extreme passing distance test.

No.	Setting Width	Unilateral Limit Distance	Results
1	0.9 m	0.03 m	Failed
2	1.0 m	0.08 m	Failed
3	1.1 m	0.13 m	Pass
4	1.2 m	0.18 m	Pass
5	1.3 m	0.23 m	Pass

## Data Availability

The datasets presented in this article are not readily available because of time and experimental site environment limitations. Requests to access the datasets should be directed to the author at youshaoze@cumt.edu.cn.
